# Beneficial soil-borne bacteria and fungi: a promising way to improve plant nitrogen acquisition

**DOI:** 10.1093/jxb/eraa112

**Published:** 2020-03-11

**Authors:** Alia Dellagi, Isabelle Quillere, Bertrand Hirel

**Affiliations:** 1 Institut Jean-Pierre Bourgin, INRAE, AgroParisTech, Université Paris-Saclay, Versailles, France; 2 Nanjing Agricultural University, China

**Keywords:** Agroecology, arbuscular mycorrhizal fungi, bacterial diazotrophs, beneficial microbes, nitrogen fertilization, nitrogen use efficiency, symbiosis

## Abstract

Nitrogen (N) is an essential element for plant productivity, thus, it is abundantly applied to the soil in the form of organic or chemical fertilizers that have negative impacts on the environment. Exploiting the potential of beneficial microbes and identifying crop genotypes that can capitalize on symbiotic associations may be possible ways to significantly reduce the use of N fertilizers. The best-known example of symbiotic association that can reduce the use of N fertilizers is the N_2_-fixing rhizobial bacteria and legumes. Bacterial taxa other than rhizobial species can develop associative symbiotic interactions with plants and also fix N. These include bacteria of the genera *Azospirillum*, *Azotobacter*, and *Bacillus*, some of which are commercialized as bio-inoculants. Arbuscular mycorrhizal fungi are other microorganisms that can develop symbiotic associations with most terrestrial plants, favoring access to nutrients in a larger soil volume through their extraradical mycelium. Using combinations of different beneficial microbial species is a promising strategy to boost plant N acquisition and foster a synergistic beneficial effect between symbiotic microorganisms. Complex biological mechanisms including molecular, metabolic, and physiological processes dictate the establishment and efficiency of such multipartite symbiotic associations. In this review, we present an overview of the current knowledge and future prospects regarding plant N nutrition improvement through the use of beneficial bacteria and fungi associated with plants, individually or in combination.

## Teaser

An updated review of how plant-associated bacterial diazotrophs and fungal endophytes may improve plant N nutrition, with an emphasis on the underlying mechanisms and their exploitation for reducing the use of chemically synthesized N fertilizers.

## Introduction

After the mid-20th century, world agriculture benefited from unprecedented changes in agronomic practices known as the “Green Revolution”. Consequently, over a 50-year period, there was a 100% increase in the yield of main crops per capita, particularly in some regions of the world such as Asia and South America (Lassaletta *et al.*, 2016; Pretty, 2018). This increase was mainly due to the technological and scientific advances over this period: new crop varieties were bred, inorganic fertilizers and chemically synthesized pesticides and herbicides were developed and extensively used, and their application was greatly facilitated by the modernization of agricultural machinery (Lassaletta *et al.*, 2016; Pretty, 2018). In particular, the use of synthetic inorganic nitrogen (N) fertilizers has increased several fold during the past 50 years (Lassaletta *et al.*, 2016; Pretty, 2018). N fertilizers chemically produced via the industrial Haber–Bosch process for agricultural purposes use 1–2% of the world’s fossil fuel energy output (Chen *et al.*, 2018a). However, because crops do not take up more than 30–50% of the N available in the soil (Wang *et al.*, 2018), the extensive use of N fertilizers has caused major detriments to microbial, animal, and plant biodiversity and to the corresponding non-agricultural ecosystems (Schlesinger, 2009; Withers *et al.*, 2014).

Cereal grains provide 60% of the food necessary to feed the world’s population, either directly as part of the human diet or indirectly as animal feed (Hirel *et al.*, 2007; Lafiandra *et al.*, 2014; Landberg *et al.*, 2019). Cereals such as maize require large inputs of N fertilizers (nitrates in particular) to achieve optimum performance. Achieving optimal economic return in an environmentally friendly manner requires that these crops use N efficiently. N use efficiency (NUE) is most commonly defined as the grain or biomass yield obtained per unit of available N in the soil (Xu *et al.*, 2012; Han *et al.*, 2015; Li *et al.*, 2017; Hawkesford and Griffiths, 2019). Currently, there are three major targets for enhancing NUE in agriculture: (i) plant breeding (Hirel *et al.*, 2007; Amiour *et al.*, 2012; Han *et al.*, 2015; Zhang *et al.*, 2015; Hawkesford and Griffiths, 2019), (ii) employing alternative and improved agronomic practices (Hirel *et al.*, 2011; Chen *et al.*, 2011; Verzeaux *et al.*, 2017a), and (iii) utilizing the beneficial effects of microbes through the study of plant microbiota (Bulgarelli *et al.*, 2013; Reinhold-Hurek *et al.*, 2015; Reis and Teixeira, 2015; Tkacz and Poole, 2015; Mus *et al.*, 2016; Gouda *et al.*, 2018; Rosenblueth *et al.*, 2018; Cordovez *et al.*, 2019; Tao *et al.*, 2019).

It is now possible to conduct research on the nature and composition of plant microbiota with next-generation sequencing (NGS) techniques. NGS has led to the discovery of the huge diversity of microbe communities present inside (endophytic communities) and on the surface of (epiphytic communities) plant organs, as well as in the rhizosphere (Bulgarelli *et al.*, 2013; Reinhold-Hurek *et al.*, 2015; Cordovez *et al.*, 2019). These microbes play several roles during plant development and are precious allies for crop production and adaptation to various biotic and abiotic stresses (Hardoim *et al.*, 2015; Tkacz and Poole, 2015; Liu *et al.*, 2017). In particular, interactions with rhizospheric and endophytic microorganisms can enhance plant mineral nutrition (Jacoby *et al.*, 2017). For instance, phosphate can be solubilized by organic acids or phytases secreted by soil-borne bacteria or fungi, thus favoring its uptake by plants (Sharma *et al.*, 2013; Alori *et al.*, 2017). Siderophores are low-molecular-weight molecules of microbial origin with a high affinity for iron. They are primarily released to solubilize iron for microbial needs, but consequently make this element more accessible to plants (Lemanceau *et al.*, 2009; Ahmed and Holmström, 2014). Regarding N, microbes play major roles in plant mineral nutrition; these roles are diverse, complex and not fully characterized, particularly during the recycling of inorganic N to mineral or gaseous forms (Coskun *et al.*, 2017). Plants can directly take up the different forms of N, such as nitrate (NO_3_^–^), ammonium (NH_4_^+^), and amino acids, by means of specific transporters (Krapp *et al.*, 2014; Fan *et al.*, 2017; Tegeder and Masclaux-Daubresse, 2018; Wang *et al.*, 2018; Hirel and Krapp, 2020). Indirect uptake of N can also occur following the establishment of beneficial associations with microbes that facilitate plant N acquisition (Santi *et al.*, 2013; Udvardi and Poole, 2013; Courty *et al.*, 2015; Chen *et al.*, 2018b).

In this review, we provide an overview of the major steps involved in N acquisition by plants, with a particular emphasis on the contribution of beneficial microorganisms that extract N from the soil or fix dinitrogen (N_2_) from the atmosphere before its transfer to the plant. Possible synergistic interactions between these microorganisms is also discussed, as it may offer an avenue for the improvement of plant productivity through the enhancement of N acquisition (Giovannini *et al.*, 2020).

## Nitrogen uptake and assimilation in plants

N is present in the soil in the form of NO_3_^–^, NH_4_^+^, or amino acids, with the availability of the various forms depending upon physical factors such as pH and temperature. Plants such as rice, which are adapted to acidic pH and anaerobic conditions, preferably take up NH_4_^+^ (Tabuchi *et al.*, 2007). In contrast, plants that are adapted to more alkaline pH in aerobic soils, as occurs in most arable lands, use mostly NO_3_^–^ as their N source (Hirel *et al.*, 2007; Masclaux-Daubresse *et al.*, 2010; O’Brien *et al.*, 2016; Xu, 2018). NO_3_^–^ is taken up by the roots and then transported in the plant by means of low- and high-affinity transporters that are, for the most part, located in the plasma membrane of cells (Krapp *et al.*, 2014; Léran *et al.*, 2014; O’Brien *et al.*, 2016; Kant, 2018; Wang *et al.*, 2018; Zhang *et al.*, 2018). Members of the nitrate transporter 1/peptide transporter family (NRT1/NPF) act as low-affinity NO_3_^–^ transporters (Fan *et al.*, 2017; Wang *et al.*, 2018). There are two exceptions in the NPF family that have been identified so far: the Arabidopsis NRTNPF6.3/NRT1.1 and the rice NRT1.1B, which display both low and high affinity for NO_3_^–^ (Ho *et al.*, 2009; Bouguyon *et al.*, 2015). High-affinity NO_3_^–^ transport systems involve transporters belonging to the NRT2 family, which belongs to the major facilitator superfamily (MFS) (Krapp *et al.*, 2014; Léran *et al.*, 2014; O’Brien *et al.*, 2016; Kant, 2018; Wang *et al.*, 2018; Zhang *et al.*, 2018). Two other families of transporters are involved in intracellular NO_3_^–^ transport: the chloride channel (CLC) family and the slow-type anion channels (SLAC) (Zifarelli and Pusch, 2010; Krapp *et al.*, 2014; Hedrich and Geiger, 2017; Wang *et al.*, 2018).

Following uptake, NO_3_^–^ is reduced to nitrite (NO_2_^–^) by the enzyme nitrate reductase (NR) located in the cytosol. Then, NO_2_^–^ is further reduced to NH_4_^+^ by a plastidic nitrite reductase. The resulting NH_4_^+^ is used to synthesize glutamine using glutamate as a substrate (Masclaux-Daubresse *et al.*, 2010; Wang *et al.*, 2018). NH_4_^+^ can also be taken up by plants directly from the soil. However, its availability mainly depends on the soil characteristics (Nieder *et al.*, 2011). Ammonium can be present in its neutral form (ammonia; NH_3_) or its cationic form (NH_4_^+^) depending on the pH of the soil or growth medium; the latter is predominant under most environmental or growth conditions (Ludewig *et al.*, 2007). When used as the sole N source, NH_4_^+^ generally induces physiological and growth perturbations in most plant species (Marino and Moran, 2019). Nevertheless, NH_4_^+^ can be directly taken up by means of high-affinity NH_4_^+^ transporters (AMT) and non-saturable low-affinity transporters (aquaporin or cation channels) (Tegeder and Masclaux-Daubresse, 2018). NH_4_^+^ transport is mediated by proteins belonging to the AMMONIUM TRANSPORTER/METHYLAMMONIUM PERMEASE RHESUS (AMT/MEP/Rh) family, located in the root cell plasma membrane. These proteins allow the transport of NH_4_^+^ to the different parts of the plant (Ludewig *et al.*, 2007; Yuan *et al.*, 2007; Tegeder and Masclaux-Daubresse, 2018). NH_4_^+^ derived from NO_3_^–^ reduction or from direct uptake is finally incorporated into organic molecules via the combined activity of two enzymes, glutamine synthase (GS) and glutamate synthase (GOGAT) (Masclaux-Daubresse *et al.*, 2010; Krapp *et al.*, 2014; Wang *et al.*, 2018; Hirel and Krapp, 2020).

The processes associated with the metabolization and recycling of N are referred to as assimilation and remobilization, respectively. N transport, assimilation, and remobilization contribute to plant NUE (Hirel *et al.*, 2007; Lea and Miflin, 2011), of which the two main components are N uptake efficiency (NupE) and N utilization efficiency (NutE) (Han *et al.*, 2015; Li *et al.*, 2017). This implies that both NupE and NutE need to be considered individually or together to improve NUE, which largely depends on the species, the genotype, and the environmental conditions. Intensive research has thus been conducted over the past three decades to find ways to improve NUE, mostly using transgenic plants or mutants altered for the expression of genes involved in N transport and assimilation (Han *et al.*, 2015; Li *et al.*, 2017; Wang *et al.*, 2018). However, these approaches generally have not led to marked and reproducible positive effects on plant growth and development. Moreover, the positive effects that have been achieved have rarely been confirmed, particularly when plants are grown under agronomic conditions (Plett *et al.*, 2017, Hirel and Krapp, 2020; Cañas *et al.*, 2020).

Due to the limited success of such approaches in terms of agronomic applications, as well as the restrictions regarding the use of genetically modified plants in many countries and the growing importance of sustainable agriculture practices, research has been reoriented to evaluate the impact of beneficial microbes on plant N acquisition (Han *et al.*, 2015; Tao *et al.*, 2019). In line with these changes in research strategies, reports suggest that plant NUE can be a key determinant favoring the establishment of beneficial microbes in the rhizosphere of rice (Zhang *et al.*, 2019). Using an elegant genetic and metagenomics approach, Zhang *et al.* (2019) showed that the rice NRT1.1b nitrate transceptor is associated with the recruitment of bacterial taxa that display enzymatic functions with high N-cycling potential. Thus, the use of beneficial microbes appears to be an attractive alternative to genetic engineering for the implementation of new breeding strategies, although they require several years of development before they can be validated and marketed.

## Plant nitrogen nutrition mediated by beneficial microbes

Microbes play essential roles in the conversion of different forms of N. These different forms of N can be used by plants following chemical reactions carried out by living organisms, and contribute to the N cycle that occurs between the soil and the atmosphere. These reactions involve bacteria, archaea, and fungi. First, atmospheric N_2_ enters the N cycle following its reduction to NH_4_^+^, a reaction catalyzed by the enzyme nitrogenase (Nase). Nase is present in N_2_-fixing symbiotic and associative symbiotic microorganisms called diazotrophs, which are mostly represented by bacteria and archaea (Coskun *et al.*, 2017; Lehnert *et al.*, 2018a). An estimated 50% of land N originates from biological N_2_ fixation (agricultural and natural) that occurs mostly in the *Rhizobium–*legume symbiosis and generates inorganic N mainly in the form of NH_4_^+^ (Fowler *et al.*, 2013; Lehnert *et al.*, 2018a). Then, NH_4_^+^ is incorporated into carbon-containing molecules for the synthesis of amino acids or oxidized by microorganisms such as nitrifying bacteria that produce nitric oxides and nitrates (Coskun *et al.*, 2017; Lehnert *et al.*, 2018a). These oxidized forms of N can be reduced by denitrifying bacteria, archaea, or fungi that convert NO_3_^–^ to NO_2_^–^, then to nitric oxide (NO), nitrous oxide (N_2_O), and finally back to N_2_ (Fowler *et al.*, 2013; Coskun *et al.*, 2017). Although most microbes involved in the N cycle do not directly interact with plants, part of the microbial community present in the soil is associated with plant roots. These microbes can have several beneficial effects, in particular on plant growth and for the acquisition of nutrients such as N, a process that is tightly linked to their biological characteristics and their ability to fix N_2_.

## Improved plant nitrogen nutrition with diazotrophs

**Fig. 1.**
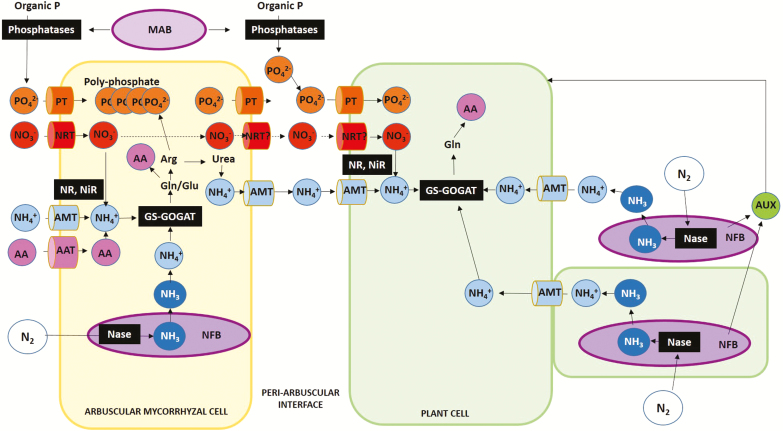
Nutritional exchanges between plant, arbuscular mycorrhizal fungi, and bacteria that help improve plant nutrition, including nitrogen (N) acquisition. Plant N acquisition can be improved in the presence of N_2_-fixing symbiotic and associative symbiotic bacteria and arbuscular mycorrhizal fungi (AMF). Minerals are acquired from the soil by the fungal extraradical mycelium and transported to the arbuscular cells. Minerals are then excreted by the fungal cells at the peri-arbuscular interface and taken up by plant cells. This transport involves specific transporters, which have not yet been fully characterized in either plants or fungi. Following solubilization by phosphatases secreted by mycorrhizae-associated bacteria (MAB), inorganic phosphate is acquired by fungal and plant cells via phosphate transporters (PT). Polyphosphate can be stored in the AMF intra- and extraradical mycelium. Polyphosphate is hydrolyzed and then inorganic phosphate is transported to the peri-arbuscular interface. Fungal cells can also take up N, ammonium (NH_4_^+^), amino acids, and small peptides. In the fungal cells, nitrate (NO_3_^–^) is reduced to NH_4_^+^ by two successive reactions catalyzed by the enzymes nitrate reductase (NR) and nitrite reductase (NiR). NH_4_^+^ is then incorporated into organic molecules via the combined action of the enzymes glutamine synthase (GS) and glutamate synthase (GOGAT), leading to the production of glutamine (Gln) and glutamate (Glu), two amino acids that foster fungal cell growth. N is finally stored in the form of arginine (Arg), which is bound to polyphosphate in the vacuolar fungal cells. Arginine is broken down into urea and then into NH_4_^+^, which is finally transferred to the peri-arbuscular interface. NH_4_^+^ is considered to be the major form of N transferred from the fungus to the plant, although it is likely that NO_3_^–^ can also be transported. Symbiotic diazotrophs such as rhizobia or *Frankia* sp. can establish highly efficient N_2_-fixing symbiosis via nitrogenase (Nase) activity and transfer N to the plant, particularly legumes, in specialized organs called nodules. A number of associative symbiotic N_2_-fixing bacteria (NFB) interact with root cells or colonize plant roots and shoots. Some of these bacteria can also secrete auxins (AUX) that promote plant growth. Diazotrophic bacteria associated with a symbiotic fungus can also contribute AMF and thus plant N acquisition. AAT, Amino acid transporter; AMT, ammonium transporter; NRT, nitrate transporter.

Biological N_2_ fixation occurs only in prokaryotes. Converting N_2_ to NH_4_^+^ is an energy-consuming process catalyzed by the multimeric enzyme Nase, which is known to be inhibited by oxygen (Fig. 1) (Hoffman *et al.*, 2014). There are two main types of symbiotic associations with N_2_-fixing bacteria (Table 1). The first involves a symbiotic association between plants and diazotrophic bacteria, leading to highly efficient atmospheric N_2_ fixation and the transfer of the fixed N to the host plant. The second type leads to much less specific and less efficient endophytic associative symbiotic interactions.

**Table 1.** Categories of interactions with microbes that can improve plant N acquisition and associated biological processes

Phylum	Family	N-associated biological process	Specificity	Efficiency of plant N nutrition improvement	Intracellular versus Extracellular	Specific cellular structure	Bacterial taxa	Plant taxa
Bacteria	Rhizobia	N fixation	High	High	Intracellular	Nodule	*Rhizobium* (alpha proteobacteria) Gram-negative	Fabaceae
	Rhizobia	N fixation	High	High	Intracellular	Nodule	*Rhizobium* (alpha proteobacteria) Gram-negative	Parsaponia sp.
	Frankia	N fixation	High	High	Intracellular	Nodule	*Frankia* sp.	Actinorhizal plants (8 taxa)
	Cyanobacteria	N fixation	Wide range	High	Intracellular/ extracellular	Heterocyst	*Nostoc* sp.	Marine, aquatic and terrestrial plants (bryophytes, pteridophytes, gymnosperms, and angiosperms)
	Other diazotrophs	N fixation	Wide range	Low/high	Intracellular/ extracellular	no	*Azospirillum* sp., *Herbaspirillum* sp*., Paenibacillus* sp*.,* etc.	Wide range including angiosperms and crops
	PGPR (including some diazotrophs)	Growth stimulation	Wide range	Low	Extracellular	no		Wide range including angiosperms and crops
Fungi	AMF	Growth stimulation/N uptake stimulation	Wide range	Low/high	Intracellular/ extracellular	Arbuscules	Glomeromycota	Wide range including angiosperms and crops

AMF, Arbuscular mycorrhizal fungi; PGPR, plant-growth-promoting rhizobacteria.

The highly efficient N_2_-fixing symbiotic associations involve bacteria belonging to the family Rhizobiaceae and Actinorhizae such as *Frankia* sp. Rhizobia are alpha-proteobacteria that establish an N_2_-fixing symbiosis mostly with legumes (Fabaceae). This is the best characterized process of endosymbiosis in plants involving N_2_-fixing bacteria. Upon molecular recognition of bacterial nodulation factors, root epidermal cells begin to differentiate into an organ called a nodule that hosts modified bacterial cells called bacteroids. Bacteroids perform N_2_ fixation in the root nodules, allowing NH_4_^+^ to be transferred to the plant via its vascular system. Biological N_2_ fixation occurring in the *Rhizobium*–legume symbiosis has been extensively reviewed (Oldroyd *et al.*, 2011; Udvardi and Poole, 2013) and will not be covered here. Some rhizobial species are able to induce the formation of N_2_-fixing root nodules in the non-legume plant *Parasponia* sp. (Behm *et al.*, 2014; van Velzen *et al.*, 2018). In this type of symbiotic association, the bacteroids are not fully differentiated as they are in legumes. In turn, both Nase activity and the number of bacterial cells per nodule are much lower compared with that in legumes (Behm *et al.*, 2014; van Velzen *et al.*, 2018). Filamentous actinobacteria of the genus *Frankia* establish symbioses with species from eight plant families belonging to the orders Fagales, Rosales, and Cucurbitales. Compared with rhizobia, the specificity of *Frankia* to their host plant is less strict (Froussart *et al.*, 2016). Interestingly, *Frankia* cells are able to fix N_2_ regardless of the symbiotic or non-symbiotic conditions, whereas rhizobia fix N_2_ only under symbiotic conditions (Santi *et al.*, 2013). *Frankia* host species are shrubs and trees growing under a large variety of environmental and climatic conditions.

Some N_2_-fixing cyanobacteria can associate with plants and provide NH_4_^+^ to their hosts without forming specialized organs such as nodules. Generally, these symbiotic N_2_-fixing cyanobacteria belong to the genus *Nostoc*. They have the capacity to differentiate specialized cells called heterocysts that carry out N_2_ fixation. These cyanobacteria can associate with a wide range of bryophytes (liverworts and hornworts), pteridophytes (*Azolla* sp.) gymnosperms (Cycadaceae), and angiosperms (Gunneraceae) (Santi *et al.*, 2013; Mus *et al.*, 2016). Cyanobacteria colonize specific structures in their hosts, which can be glands located on the dorsal lobe of leaves (*Azolla*) or cavities in stems (*Gunnera*) (Santi *et al.*, 2013). Most interactions between plants and cyanobacteria are facultative, except for *Azolla* sp., which has been used as an N biofertilizer for thousands of years in rice paddy fields (Kollah *et al.*, 2016). Many plant species can associate with associative symbiotic free-living N_2_-fixing bacteria (AS-NFB). AS-NFB are present in a number of taxa, including alpha-proteobacteria, beta-proteobacteria, gamma-proteobacteria, and firmicutes. They include *Azoarcus*, *Azospirillum*, *Herbaspirillum*, *Azotobacter*, *Burkholderia*, *Klebsiella*, *Gluconobacter*, *Acetobacter*, and *Pseudomonas* spp. (Santi *et al.*, 2013; Reinhold-Hurek *et al.*, 2015; Mus *et al.*, 2016). A number of these microorganisms are present in the rhizosphere and remain at the root surface, whereas others can colonize the plant endosphere; they are classified as plant-interacting or endophytic bacteria, respectively. Diazotrophic bacteria are present in the rhizosphere of many wild and cultivated plant species, such as cereals. Due to their ability to reduce N_2_ to NH_4_^+^, a form of N readily utilizable by the host plant, there is growing interest in determining the physiological and molecular mechanisms underlying this plant–bacterial association (Rosenblueth *et al.*, 2018). Therefore, several studies have been undertaken to isolate and characterize these diazotrophic bacteria, particularly when the associated or host plants are grown under low N fertilization conditions. The characterization of these bacteria is primarily based on the detection of *nif* gene sequences encoding Nase.

Studies have demonstrated that N_2_ fixation occurs in bacteria harboring *nif* genes and that there is a subsequent transfer of NH_4_^+^ to the plant. Nase activity can be measured using the acetylene reduction assay (ARA) and by means of ^15^N dilution experiments. The ARA is based on the capacity of Nase to reduce acetylene to ethylene, a molecule that is easily quantified by gas chromatography (Kifle and Laing, 2015; Brusamarello-Santos *et al.*, 2017). ^15^N dilution experiments can estimate the contribution of diazotrophic ^14^N_2_ fixation to total plant N acquisition following ^15^N labelling (Chalk, 2016). A few studies have shown that AS-NFB can fix N_2_ whether they are associated with or colonize the plant, and that the resulting inorganic N is transferred to the plant (Sevilla *et al.*, 2001; Hurek *et al.*, 2002; Iniguez *et al.*, 2004; Pankievicz *et al.*, 2015). To increase the efficiency of AS-NFB in terms of their ability to transfer N to plants, genetic manipulations (mutations or introduction of constitutive promoters) can be conducted to deregulate NH_4_^+^ production and excretion for direct incorporation by shoots and roots (Ambrosio *et al.*, 2017; Santos *et al.*, 2017). Wheat inoculation with a spontaneous mutant excreting large amounts of NH_4_^+^ resulted in an increase in plant yield (Santos *et al.*, 2017).

In addition to providing N to the plant, several AS-NFB have the ability to promote root and shoot growth of wheat, maize, rice, and sorghum through the production of hormones, such as auxins, known to promote organ growth (Dobbelaere *et al.*, 1999; Santi *et al.*, 2013; Vacheron *et al.*, 2013). These bacteria are referred to as plant-growth-promoting rhizobacteria (PGPR) (Cassán and Diaz-Zorita, 2016; Kudoyarova *et al.*, 2019). Because the effect of PGPR can be multifactorial, it has been described by the “Multiple Mechanisms Theory” (Bashan and de-Bashan, 2010).

In a symbiotic *Rhizobium*–legume association, the plant benefits from the increased availability of reduced N_2_, while the bacteria utilize carbohydrates provided by the host plant (Udvardi and Poole, 2013; Hoffman *et al.*, 2014). When there is an interaction between a plant and diazotrophic bacteria, the vicinity of the roots is enriched with carbohydrates present in the root exudates. It is thus likely that these carbohydrates are used to provide the energy required for N_2_ fixation. In line with this hypothesis, reports indicate that, in a tropical maize genotype, aerial roots that produce a mucilage constitute a chemoattractive niche for diazotrophic bacteria (Van Deynze *et al.*, 2018). This mucilage provides substantial amounts of carbohydrates to the bacteria, leading to a remarkably active N_2_ fixation process that is greatly favored in this sugar-enriched environment (Knee *et al.*, 2001; Van Deynze *et al.*, 2018). Future agronomic developments utilizing the ability of plant genotypes to release carbohydrates in root exudates not only to attract AS-NFB but also to boost beneficial bacterial colonization and N_2_ fixation thus requires further research (Coskun *et al.*, 2017; Jacoby *et al.*, 2017).

## Improved plant nitrogen nutrition in plant–fungus associations

Plant N acquisition can also be enhanced when plants develop symbiotic mycorrhizal associations with fungi of the order Glomeromyta by means of mycelium that colonizes root cells (endomycorrhiza) or is attached extracellularly to them (ectomycorrhiza). This type of symbiotic association makes it possible to capture nutrients in the surrounding rhizosphere. In particular, the fungi provide minerals to the host plant and improve plant tolerance to biotic and abiotic stresses (Wang *et al.*, 2017; Choi *et al.*, 2018; Ferlian *et al.*, 2018). Arbuscular mycorrhizal fungi (AMF) are the most widespread symbiotic association: they colonize 80–90% of terrestrial plants (Gianinazzi *et al.*, 2010). Following spore germination, AMF colonize intra- and inter-cellular spaces and then develop highly branched structures called arbuscules in the cortical cells of the host plant. These structures are surrounded by a peri-arbuscular interface where nutrient exchanges with the host plant cells take place. The arbuscules are connected to an intraradical mycelium that is present inside the root tissues. The intraradical mycelium is also connected to an extraradical mycelium that spreads in the soil, allowing the uptake of minerals such as phosphate and N (Fig. 1) (Chen *et al.*, 2018b; Choi *et al.*, 2018; Ferrol *et al.*, 2019).

Following colonization by AMF, plant transcripts of genes coding for NO_3_^–^ and NH_4_^+^ transporters are up-regulated (Hildebrandt *et al.*, 2002; Courty *et al.*, 2015; Garcia *et al.*, 2016; Koegel *et al.*, 2017). Similarly, fungal NO_3_^–^ and NH_4_^+^ transporters are up-regulated during the plant colonization process (Courty *et al.*, 2015; Koegel *et al.*, 2017). So far, most of the studies aiming to understand the mechanisms involved in plant N nutrition in the presence of AMF have been based on gene transcriptional activation (Guether *et al.*, 2009; Pérez-Tienda *et al.*, 2014; Garcia *et al.*, 2016; Chen *et al.*, 2018b). Interestingly, specific up-regulation of some transporters has been observed in roots colonized by AMF, indicating that under these conditions the transport mechanism is modified compared with that found in non-colonized plants (Garcia *et al.*, 2016). For instance, the *AMT3.1* plant NH_4_^+^ transporter transcript is specifically up-regulated in cereals such as sorghum, maize, and rice colonized by AMF (Koegel *et al.*, 2017). The same study proposed that AMT3.1 is the major driver of N and phosphorus (P) transfer to the plant colonized by AMF (Koegel *et al.*, 2017). Thus, the whole N uptake machinery available to the plant in the absence of mycorrhizae is not entirely recruited during these interactions.

Part of the N acquired by the fungal cells is used for their own metabolism. Storage of N as arginine (Arg) is indicated by the large amounts of Arg found in the extraradical mycelium and by the up-regulation of the genes and enzymes involved in the biosynthesis of Arg in the extraradical mycelium and its breakdown into urea in the intraradical mycelium (Cruz *et al.*, 2007; Chen *et al.*, 2018b). Arg is strongly bound to polyphosphate in vacuolar fungal cells, which may reflect a connection between P and N metabolisms (Fig. 1) (Woods and Ferré, 2005; Kikuchi *et al.*, 2014). NH_4_^+^ is considered to be the major form of N transfer from the fungus to the plant, although there may be NO_3_^–^ transfer as well because NRT genes from both the plant and the fungus have been found to be up-regulated (for review, see Courty *et al.*, 2015; Garcia *et al.*, 2016; Chen *et al.*, 2018b). Carbohydrates and long-chain fatty acids are excreted by plant cells in the peri-arbuscular interface and then acquired by fungal cells via specific transporters (MacLean *et al.*, 2017, Choi *et al.*, 2018). Further investigations are now needed to determine whether improved plant N acquisition is due to the transfer of N from the AMF or to an increase in N uptake triggered by the fungus, considering at the same time that the fungus and the plant can compete for limited resources (Makarov, 2019). Developing such studies may be a means to further assess whether AMF symbiosis has a direct or an indirect impact on plant N nutrition. In addition, it is necessary to determine whether there is competition for N acquisition between the plant and the fungus, which might imply that, under agronomic conditions, N fertilization needs to be optimized for the plant to benefit from the symbiotic association.

## Improvement in plant nitrogen nutrition by combining bacterial and mycorrhizal colonization

A promising strategy to enhance plant nutrition and thus plant performance is through the development of tripartite associations with bacteria and mycorrhizal fungi (Wu *et al.*, 2005, Artursson *et al.*, 2006; Bonfante and Anca, 2009, Giovannini *et al.*, 2020). AMF are in some cases associated with other microbes in the rhizosphere. Such associations can be either beneficial or detrimental to the AMF (Jansa *et al.*, 2013). Although these tripartite associations are not very well characterized yet, they seem to strongly depend on nutritional exchanges between AMF and bacteria. These exchanges involve the production of exudates by the fungi that attract the bacteria, and the ability of the bacteria to facilitate the access to nutrients of the fungi (Kaiser *et al.*, 2015; Zhang *et al.*, 2016). For instance, bacteria can more easily solubilize phosphate than fungi, thereby improving both fungal and plant phosphate nutrition (Zhang *et al.*, 2016). Interestingly, bacteria of the genus *Paenibacillus* isolated from the roots of *Sorghum bicolor* are able to stimulate mycorrhizal colonization of the plant by *Glomus mosseae* (Budi *et al.*, 1999). Moreover, *Paenibacillus* sp. has been detected inside cells of the ectomycorrhizal fungus *Laccaria bicolor* (Bertaux *et al.*, 2003). *Paenibacillus validus* is a species that can promote the growth *in vitro* and the formation of spores in *Rhizophagus irregularis* (Hildebrandt *et al.*, 2006). This stimulating effect on spore production may be due to the presence of sugars secreted by the bacterium that enhance fungal growth until spores form. Thus, enhanced fungal growth may be beneficial to the development of a mycorrhizal association with a plant (Hildebrandt *et al.*, 2006). In addition, a number of *Paenibacillus* species are potential N_2_ fixers that can solubilize phosphate and iron and secrete phytohormones (Grady *et al.*, 2016). Several plant-associated fungi are colonized by endosymbiotic diazotrophs that can provide N to the fungi (Minerdi *et al.*, 2001; Sharma *et al.*, 2008, Torres-Cortés *et al.*, 2015; Paul *et al.*, 2020).

These various tripartite associations can favor the establishment of more efficient fungal and N_2_-fixing symbioses, potentially leading to better acquisition of N by the plant (Paul *et al.*, 2020). Thus, further investigations to identify bacteria favoring the establishment of a symbiotic association between plants and AMF are worth conducting to determine whether this tripartite association can boost plant N acquisition, particularly under reduced fertilization conditions (Giovannini *et al.*, 2020). Associating plants with more complex microbial consortia is another strategy that can boost plant performance, because AMF inoculation in combination with a microbial consortium isolated from non-fertilized soils increase N uptake in *Brachypodium dystachion* (Hestrin *et al.*, 2019).

## Concluding remarks

To fully exploit the beneficial effect of soil microbiota to improve plant N acquisition, more research is required to study and thus optimize the associations between plants, bacteria, and fungi in order to use them for the implementation of more sustainable agricultural practices (Hirel *et al.*, 2011; Verzeaux *et al.*, 2017a). First, it will be necessary to perform large-scale surveys and experiments to isolate bacterial and fungal species or strains associated with crop species or crop varieties and determine in which area in the world improved plant performance is observed, not only under laboratory conditions but also in the field (Cassán and Diaz-Zorita, 2016). This will allow the identification of microorganisms (or combinations of microorganisms) and crop genotypes (races collected around the world, lines, hybrids, etc.) that develop efficient interactions, particularly when N fertilization is lowered or limiting.

Furthermore, there clearly is genetic diversity among crop varieties for beneficial plant–microorganism associations, which suggests that, both among different species and within the same species, there are favorable alleles involved in the control of such associations (Sawers *et al.*, 2018; Valente *et al.*, 2020, Chandra *et al.*, 2020). More ambitious studies at the genome level could be undertaken to identify which genes or loci are involved in the genetic control of plant performance in response to inoculation with N_2_-fixing bacteria and AMF (Lehnert *et al.*, 2018b; Vidotti *et al.*, 2019).

**Fig. 2.**
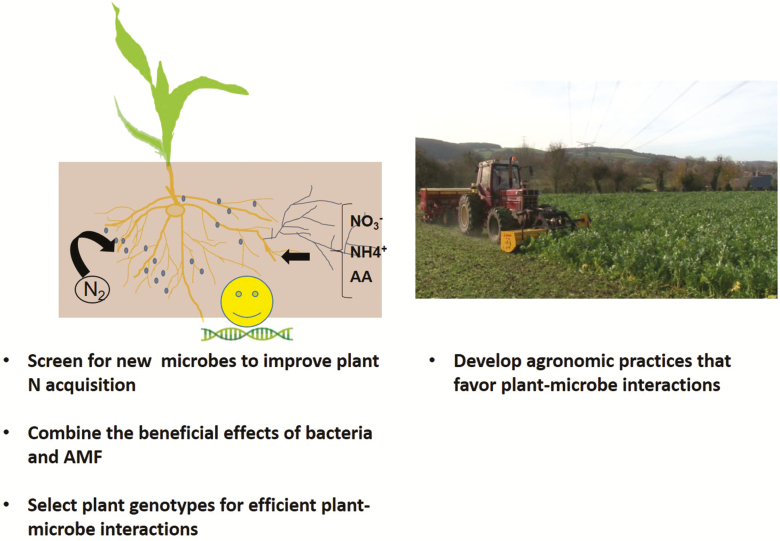
Strategies to optimize interactions with beneficial microbes to improve plant nitrogen (N) acquisition. Various strategies can be combined to reduce the use of N fertilizers and improve crop N acquisition. They include (i) capitalizing on the beneficial impact of microbes either individually or when they are present in tripartite associations; (ii) utilizing plant genetic deversity to select the most efficient interactions in terms of the acquisition of nutrients, N in particular; and (iii) developing agronomic practices such as no-till and those based on the use of cover crops that favor plant–microbe interactions (Hirel *et al.*, 2011).

Another important issue will be to assess the persistence and competitiveness of beneficial microbes when there is a positive interaction with plants, particularly when microbial inoculants are used to improve plant performance (Romano *et al.*, 2020). In parallel, fundamental research needs to be conducted to characterize the interactions regarding N acquisition between plants and beneficial microorganisms at the physiological and molecular levels. This type of study can help identify markers that can further help breeders to screen or develop new crop varieties for their responsiveness to microorganisms, either already present in the rhizosphere (van der Heijden *et al*., 2008) or provided as bio-inoculants. The agronomic practices adapted to or developed for these new varieties must be “microbe friendly” so that they favor and do not reduce soil-borne beneficial microbes (Fig. 2) (Hirel *et al.*, 2011; Verzeaux *et al.*, 2017a). Because fungicides are detrimental to soil microbial populations, the reduction in pesticide use can promote the benefits of soil microbiota ecosystemic services (Jacobsen and Hjelmsø, 2014). No-till farming, which is based on sowing seeds without disturbing the soil, is being adopted by growers because it allows the conservation of mycorrhizal hyphal soil networks (Verzeaux et al., 2017a, b). The use of cover crops, grown between the main crop, mown, and then left in place before sowing the next crop, helps conserve soil organic matter, favoring soil microbe proliferation (Verzeaux *et al.*, 2016). A wise combination of all these strategies will help reduce the use of N fertilizers (Verzeaux *et al.*, 2017a).
